# Ethnic diversity and mortality in northwest Burkina Faso: An analysis of the Nouna health and demographic surveillance system from 2000 to 2012

**DOI:** 10.1371/journal.pgph.0000267

**Published:** 2022-05-06

**Authors:** Zahia Wasko, Peter Dambach, Gisela Kynast-Wolf, Gabriele Stieglbauer, Pascal Zabré, Cheik Bagagnan, Anja Schoeps, Aurélia Souares, Volker Winkler

**Affiliations:** 1 Institute of Global Health, University Hospital Heidelberg, Heidelberg, Baden Württemberg, Germany; 2 Health & Demographic Surveillance System, Centre de Recherche en Santé de Nouna (CRSN), Nouna, Burkina Faso; FIOCRUZ: Fundacao Oswaldo Cruz, BRAZIL

## Abstract

Ethnic diversity has been a topic of contention across the globe, contrasted with economic development, social security, and political stability. The link between health and ethnic diversity is not yet well established especially in low-middle- income countries. Our study aims to explore the association between ethnic diversity and all-cause mortality in rural areas of Burkina Faso. We used data from the Nouna Health & Demographic Surveillance System (HDSS) collected between 2000 and 2012. To derive Standardized Mortality Ratios (SMR), the observed number of deaths was compared to the expected deaths based on the entire HDSS taking into account sex, age, rainy season, calendar year, and village. SMR were calculated for ethnic and religious diversity on a village level (using the Simpson Index), sub-region, wealth, and distance to Healthcare Facilities (HCF). Furthermore, we modeled SMR with a multilevel random intercept Poisson regression considering individual ethnic and religious groups in addition to the above-mentioned village-level information. Village wealth (poorest fifth: SMR 1.07; 95% CI: 1.02–1.13, richest fifth: SMR 0.85; 95% CI: 0.82–0.88), distance to HCF (within the village: SMR 0.88; 95% CI: 0.85–0.91, further than 5km: SMR 1.13; 95% CI: 1.10–1.16), and sub-region showed significant associations with overall mortality. Villages belonging to the third with the highest ethnic diversity had lowered SMR (0.86; 95% CI: 0.84–0.89) compared to the entire HDSS, while those belonging to the lowest diversity third yielded elevated SMR (1.13; 95% CI: 1.09–1.17). The multilevel model confirmed the association. Our study showed that historically established ethnic diversity in rural areas of Burkina Faso was associated with lower all-cause mortality. Generally, the literature suffers from a lack of standardization in defining ethnic diversity, along with measuring it. More research is needed to understand this relation and to establish it in different settings.

## Introduction

Throughout the history, ethnic diversity has been highlighted as a heated topic when debating core issues such as economic development, taxation, and political stability. Scholars are increasingly studying ethnic diversity and its effects in societies on economic, social, political, and health outcomes [1–8]. Recent studies from different countries showed a complex picture with positive effects of ethnic diversity on social cohesion, trust, and provision of goods [1–3, 8]. For example, a study conducted on neighborhoods across Great Britain, concluded that ethnic diversity can negatively affect social trust only if associated with medium to high levels of segregation [4]. Another study analyzing 10-year data from 35 countries in Sub-Saharan Africa (SSA), showed that higher levels of country ethnic diversity led to more developed financial systems [5].

The link between health and ethnic diversity is not yet well founded and so far, research exhibits contradicting effects. For instance, data from a cross-section of countries demonstrated that higher levels of ethnic diversity were linked to poorer health outcomes including less immunization uptake, higher prevalence of the human immunodeficiency viruses and malaria, in addition to higher mortality rates, and poorer health infrastructure [6]. On the other hand, the same investigator led an analysis in India, suggesting a positive effect of ethnic diversity on health indicators, such as child mortality and education [9]. A recent cohort of neighborhoods in England showed that an initial exposure to higher ethnic diversity was negatively associated with subjective well-being; whereas long-term exposure had no effect [10].

Burkina Faso is part of a diverse region in SSA inhabited by many different ethnic groups historically co-existing. In the Northwestern part lies the rural province of Kossi. Its population is young, with 46% being less than 15-year-old. The main activities remain by far subsistence farming and stock breeding. In 2018, the percentage of people living below the poverty line was 41.8% while the fraction of literacy was 41.2% [11]. The ethnic groups Dafing, Bwaba, Mossi, and Peuhl represent 90% of all individuals. Across ethnic groups, people follow various religions and rites such as: Islam, Catholicism, Protestantism, and Animism [12]. Burkina Faso is unique among Western African countries because ethnic groups are well dispersed geographically, and religious diversity is found in every ethnic group [13]. This geographic dispersion is historic and is related to extensive trading networks, armed conflicts, and colonial conquest [14].

Mortality, as a proxy for general health, has been studied for long in the Kossi province to understand health issues including malaria, child health, perinatal health, and overarching access to healthcare services [15–19]. An overall improvement in mortality rates throughout the last decades is noticeable; however, compared to global rates, the mortality is still among the highest [20, 21]. Previous research drew associations between death rates and other contextual factors such as the accessibility to healthcare and socio-economic position (SEP) of households [22–24]. Yet, no study looked at ethnic and religious composition of the villages and their possible association with health. Our study aims to reduce the gap in literature on ethnic diversity and health by exploring the association between ethnic diversity and all-cause mortality. The diversity construct is complex: it has been defined differently across and within disciplines [25]. In this study, as we are interested in the concept of living in an ethnically diverse context, we considered ethnic diversity the extent of variety of ethnic groups within villages. This reflects the contribution of all ethnic groups represented within a village to define its level of diversity. Whereas other methods, example the majority-minority approach, focus simply on certain ethnic groups within a defined context (minority groups versus majority). These approaches overlook the relative group proportion in the overall composition of the defined context [25, 26].

## Methods

The study is founded on data derived from the Health and Demographic Surveillance System (HDSS) of Nouna, managed by the Nouna Health Research Center in the Kossi province, Northwest Burkina Faso. The region has a Sudano-Sahelian climate characterized by a rainy season between June and September [27]. The Nouna HDSS is a dynamic prospective cohort that regularly collects data from households in the defined study area on events, such as births, deaths, and migration [12]. During the observation period from January 2000 until December 2012, the HDSS covered an area of 1,775 km^2^ counting 58 villages, as well as the semi-urban town of Nouna. The total population was about 80,000 individuals living in more than 10,000 households [28]. The study area included 16 basic Healthcare Facilities (HCF) called CSPS (Centre de Santé et de Promotion Sociale) and one hospital referred to as CMA (Centre Médical avec Antenne chirurgicale) [29]. Ten of those 16 HCF were inaugurated between 2004 and 2011 and for the analysis, the year of inauguration was considered.

The permission to conduct this study was granted by the Nouna Health Research Center in Burkina Faso and the Heidelberg Institute of Global Health in Germany. There were no case presentations that required disclosure of respondents’ confidential data/information in this study. Additional information regarding the ethical, cultural, and scientific considerations specific to inclusivity in global research is included in the Supporting Information (S1 Checklist).

All-cause mortality was analyzed utilizing Standardized Mortality Ratios (SMRs) calculated by the ratio of the observed and the expected number of deaths. The expected number was derived from the product of the mortality rates observed in the entire HDSS and the accumulated Person-years (PY) split by sex, 5-year age group, calendar year, rainy season, and village. For the regression analysis, data was additionally split by ethnic groups (Bwaba, Dafing, Mossi, Peuhl, and “other”, including less common ethnic groups and missing information (2%)), as well as religious groups (Animist, Christian, Muslim, and “other”, including less common religious groups and missing information (<1%)).

We calculated SMRs with the exact 95% Confidence Intervals (CI) according to the following village level characteristics: (i) sub-region (West, North-East, South-East, and Nouna town); (ii) distance to the next HCF (within the village, <5 km, ≥5 km); (iii) household-based mean village wealth (quintile cut-offs) [23], (iv) religious diversity (tertiles), and (v) ethnic diversity (tertile cut-offs).

To define the village wealth, we adapted a household wealth indicator created for each household in the study area from the year 2009. This indicator was derived utilizing the Principal Components Analysis (PCA), described in detail elsewhere [23]. The village wealth was defined as the mean household wealth for every village.

In general, diversity is measured differently across studies and disciplines. One common approach is the Diversity Index, also called, the Simpson Index [30]. Even though different equations exist, all variations quantitatively reflect the degree of concentration of individuals in a community, when classified into e.g., ethnic groups. We estimated the mean ethnic and religious diversity of each village considering the groups mentioned above, for the whole observation period, according to the Simpson Index with the formula:

$$D=1-\sum_{i=1}^{g}p_{i}^{2}$$

where D denotes the index, p conveys the fraction of a group i, and g indicates the number of groups present in the respective village [30]. The index ranges from 0 to 1 and equals the probability that two random individuals of a same village belong to different groups.

**Fig 1:**
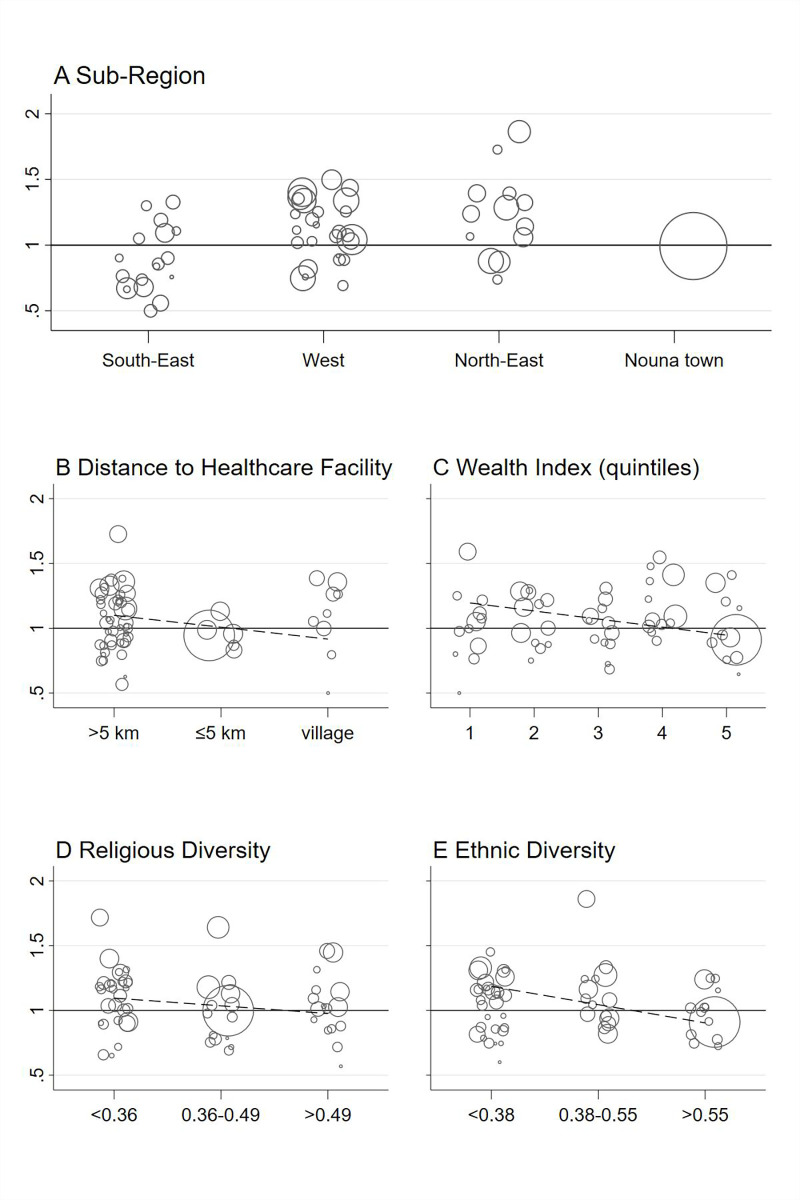
Standardized mortality ratios of each village by village characteristics. SMRs are calculated in comparison to the entire HDSS, the bubble size indicates the number of observed deaths, dashed lines indicate linear trends (weighted by the number of observed deaths).

**Fig 2:**
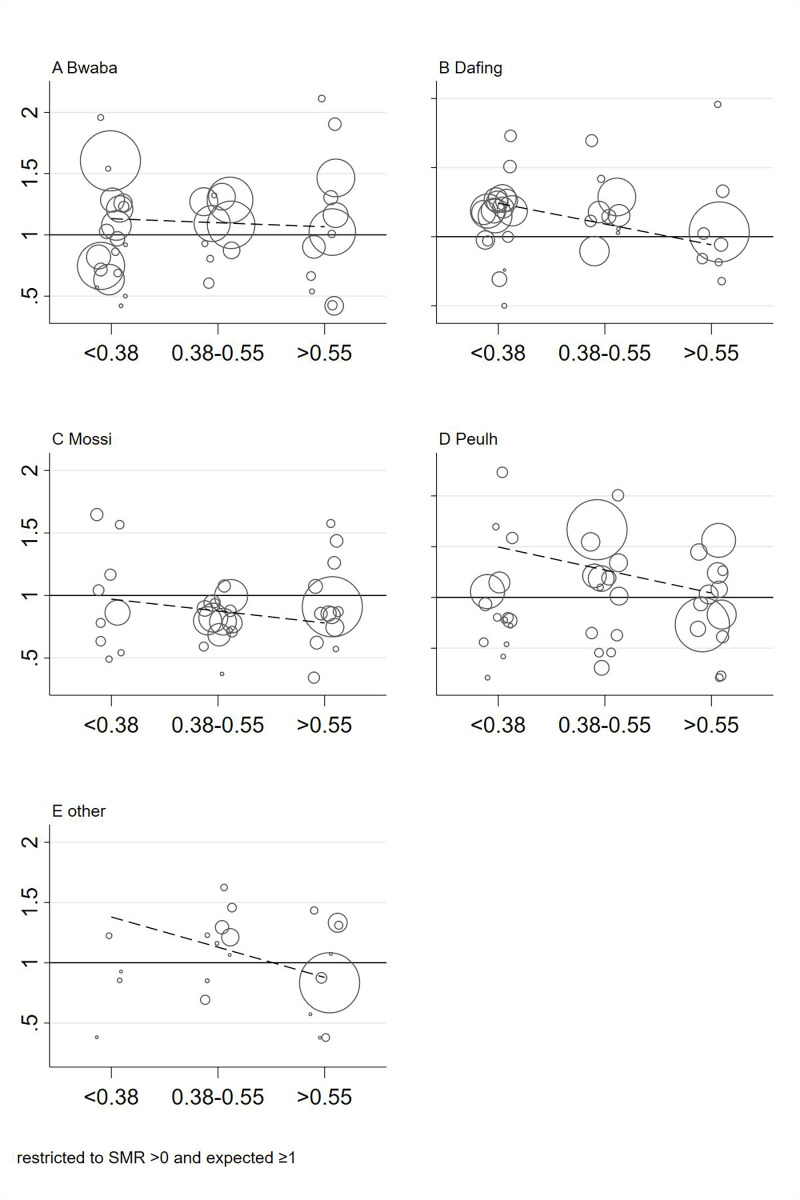
Standardized mortality ratios of each village by ethnic diversity separated for ethnic groups. SMRs are calculated in comparison to the entire HDSS, the bubble size indicates the number of observed deaths; dashed lines indicate linear trends (weighted by the number of observed deaths).

We plotted the SMR of each village against the village characteristics in scatter plots and added linear trends weighted by the number of observed deaths (Fig 1). Additionally, we plotted the SMR against ethnic diversity thirds separately for each ethnic group to depict the consistency of the association across ethnic groups (Fig 2). In Figs 1 and 2, villages with zero observed deaths or less than 1 expected death were not plotted to improve visibility.

In a final step, we modeled the SMR using a multilevel Poisson regression with random intercepts considering two levels of information (Table 3). The number of observed deaths was the dependent variable, and the log of the expected deaths was used as the offset. Independent variables were all entered categorically as given above. The first level (individual) accounted ethnic and religious groups, while the second level (village) accounted region, distance to HCF, wealth, religious diversity, and ethnic diversity. Standard errors were controlled for overdispersion [31]. Statistical analyses were performed using Stata/SE 15.1 for Windows (64-bit x86-64) Revision 26 Aug 2019 (StataCorp LLC, 4905 Lakeway Drive, College Station, TX 77845, USA).

## Results

During the observation time of 13 years, a total of 10,137 deaths was observed in the Nouna HDSS with 1,016,085.8 PY. On average, 78,160 people were observed. Table 1 depicts the sample characteristics showing crude mortality rates for all information used to split the PY, except for the village-specific rates given in S1 Table. There are considerable differences with respect to all characteristics and, naturally, the highest exists for age. The shown decrease in mortality over time is known [21, 29]. However, the relatively strong differences between religious groups have not been described sufficiently. Furthermore, there are noticeable differences in mortality between ethnic groups.

**Table 1:** Sample characteristics of the Nouna Health and Demographic Surveillance System from 2000–2012.

	Observed deaths	Person-Years	Crude mortality rate per 1,000 (95% CI)
Total	10,137	1,016,085.8	10.0 (9.8–10.2)
Sex			
Male	5,284	509,653.5	10.4 (10.1–10.7)
Female	4,853	506,432.3	9.6 (9.3–9.9)
Age			
< 5 years	4,472	179,650.1	24.9 (24.2–25.6)
05–29 years	1,221	562,572.6	2.2 (2.1–2.3)
30–54 years	1,233	196,460.7	6.3 (5.9–6.6)
> 54 years	3,211	77,402.4	41.5 (40.1–42.9)
Calendar year			
2000–2004	4,739	407,212.7	11.6 (11.3–12.0)
2005–2008	2,438	248,517.0	9.8 (9.4–10.2)
2009–2012	2,960	360,356.1	8.2 (7.9–8.5)
Season			
Dry	5,983	590,297.9	10.1 (9.9–10.4)
Rainy	4,154	425,787.9	9.8 (9.5–10.1)
Ethnic group			
Bwaba	2,448	244,193.9	10.0 (9.6–10.4)
Dafing	4,295	384,427.7	11.2 (10.8–11.5)
Mossi	1,478	183,966.4	8.0 (7.6–8.5)
Peuhl	952	92,168.7	10.3 (9.7–11.0)
Other	964	111,329.2	8.7 (8.1–9.2)
Religious group			
Animist	941	57,400.3	16.4 (15.4–17.5)
Christian	2,690	320,094.3	8.4 (8.1–8.7)
Muslim	6,481	635,988.6	10.2 (9.9–10.4)
Other	25	2,602.6	9.6 (6.2–14.2)

Table 2 shows the indirectly standardized ratios between the observed and the expected numbers of deaths based on the entire Nouna HDSS, accounting for sex, age group, calendar year, and season. SMRs for the village characteristics show substantial differences. The differences between sub-regions are strong, showing the highest ratio for the Western region (SMR 1.12 (95% CI 1.08–1.15) and the lowest for Nouna town (SMR 0.83 (95% CI 0.79–0.86)). As expected, and studied previously, the distance to the next HCF significantly affects the overall mortality, resulting in a lowered ratio of 0.88 (95% CI 0.85–0.91) when a facility is available within a village [32]. With respect to the mean wealth of the village, a clear effect could only be seen for the richest fifth with an SMR of 0.85 (95% CI 0.82–0.88). Religious diversity shows an unclear picture for increasing diversity with the highest ratio for the least diverse third. In contrast, increasing ethnic diversity is associated with decreasing mortality. Each third with higher ethnic diversity results in a significantly lower SMR from 1.13 (95% CI 1.09–1.17), to 1.06 (95% CI 1.02–1.10), to 0.86 (95% CI 0.84–0.89).

**Table 2:** Standardized mortality ratios for village characteristics in comparison to the entire Nouna Health and Demographic Surveillance System.

	Observed deaths	Expected deaths	SMR (95% CI)
Sub-region			
West	3,903	3,489.5	1.12 (1.08–1.15)
North-East	2,163	1,864.3	1.16 (1.11–1.21)
South-East	1,553	1,734.9	0.90 (0.85–0.94)
Nouna town	2,518	3,048.3	0.83 (0.79–0.86)
Distance to healthcare facility			
Within the village	4,530	5,138.2	0.88 (0.85–0.91)
<5km	1,493	1,362.8	1.10 (1.04–1.15)
≥5km	4,114	3,636.0	1.13 (1.10–1.16)
Wealth index (fifths)			
1^st^ (poorest)	1,669	1,553.3	1.07 (1.02–1.13)
2^nd^	2,023	1,844.2	1.10 (1.05–1.15)
3^rd^	1,618	1,516.0	1.07 (1.02–1.12)
4^th^	1,447	1,233.6	1.17 (1.11–1.24)
5^th^	3,380	3,989.9	0.85 (0.82–0.88)
Religious Diversity (0–0.64)			
< 0.36 (less diverse)	3,367	3,055.5	1.10 (1.07–1.14)
0.36–0.49	4,758	5,135.0	0.93 (0.90–0.95)
> 0.49	2,012	1,946.4	1.03 (0.99–1.08)
Ethnic Diversity (0.01–0.77)			
< 0.38 (less diverse)	3,560	3,148.0	1.13 (1.09–1.17)
0.38–0.55	2,942	2,782.4	1.06 (1.02–1.10)
> 0.55	3,635	4,206.6	0.86 (0.84–0.89)

The expected number of cases accounted for sex, age group, calendar year, and season.

Fig 1 also shows the SMRs for all village-level characteristics separately for each village. The bubble size is proportional to the observed number of deaths. All characteristics, with a logical order (Fig 1B–1E), include a correlation line. Fig 2 separates the correlation of all-cause mortality and ethnic diversity shown in Fig 1E by the different ethnic groups. Except for Bwaba, there was a negative correlation; meaning that lower mortality was associated with increased ethnic diversity.

Table 3 presents the results of the multilevel Poisson regression, modelling the standardized mortality ratio for the village characteristics (level 2) while considering religious groups, along with ethnic groups (level 1). The regression coefficients are expressed as relative ratios in comparison to the respective reference category. The level 1 coefficients reflect the results from the crude rates shown in Table 1. The same is true for the level 2 variables except for the distance to the next HCF. Looking particularly at ethnic diversity, the lowest relative ratio was observed for the highest third of diversity, while the other two thirds were not different from each other.

**Table 3:** Multilevel Poisson regression of the standardized mortality ratio with random intercepts on the village level.

	Exp (coefficient)	95% CI	P-value
level 1 (individual)			
Ethnic group			<0.001
Bwaba	0.97	0.89–1.06	
Dafing	1*		
Mossi	0.80	0.75–0.86	
Peuhl	0.90	0.82–0.97	
other	0.92	0.85–0.99	
Religious group			<0.001
Animist	1.22	1.12–1.34	
Christian	0.93	0.87–0.99	
Muslim	1*		
other	1.02	1.12–1.34	
Level 2 (village)			
Sub-region			0.001
West	1.19	1.08–1.31	
North-East	1.25	1.08–1.44	
South-East	1*		
Nouna town	1.19	1.01–1.40	
Distance to healthcare facility			<0.001
within the village	0.94	0.86–1.03	
≤5 km	0.87	0.80–0.93	
>5 km	1*		
Wealth index (fifths)			0.006
1^st^ (poorest)	1*		
2^nd^	0.93	0.84–1.03	
3^rd^	0.98	0.88–1.10	
4^th^	1.12	0.99–1.26	
5^th^	0.91	0.80–1.03	
Religious Diversity (0–0.64)			0.593
< 0.36 (less diverse)	1*		
0.36–0.49	1.05	0.94–1.17	
> 0.49	1.06	0.94–1.19	
Ethnic Diversity (0.01–0.77)			0.093
< 0.38 (less diverse)	1*		
0.38–0.55	1.01	0.93–1.12	
> 0.55	0.90	0.81–1.01	

* reference category

## Discussion

This study analyzed the relationship between the level of ethnic diversity of villages in a rural West-African setting and all-cause mortality as a contribution to the discussion around the potential link between ethnic diversity and health. We found an association between high levels of ethnic diversity and decreased mortality, which remained stable after controlling for individual level, as well as community level information.

An ethnological analysis of Burkina Faso that explored joking behaviors in the culture might help explain our results. It highlighted the crosscuttings identities among ethnic groups generally in West Africa and more specifically in Burkina Faso. The ethnographer explained that ethnic groups in this region originated from clans and family ties. However, after ethnic dispersion occurred, clans in hosting communities allowed refugees and newcomers from other ethnic groups to adopt their names in a step to integrate them and incorporate them in the system. This practice further strengthened social cohesion between ethnic groups since belonging to an ethnic group was defined beyond kinship [14]. Another anthropological, political analysis of Burkina Faso highlighted the community-oriented lifestyle and the interdependence living as a necessity for survival, especially in harsh and poor conditions, usually found in rural areas [13]. This interdependence, or social cohesion, when combined with ethnic diversity may lead to individual empowerment, willingness to challenge social norms, as well as improved education [33–35]. All those factors are known to improve health [34, 36, 37]. Also, the unique ethno-religious setting of Burkina Faso where ethnicity and religion are hardly intertwined may create high levels of tolerance and openness toward the other, translated into high interactions among the different religious and ethnic groups [13]. High interactions between different ethnic groups may also increase exposure, acceptance, and even adoption of differing ideas and beliefs [38]. These consequences bring about an inclusive environment, empowering individuals; for instance, to face their illness and seek treatment without fear of stigmatization.

Lastly, ethnic diversity may lead to a community economic improvement as it comes hand in hand with resource diversity, especially in manpower and market composition [8]. The positive effect of improved SEP in communities on health is well established in the literature [39–42].

In general, our findings come in-line with results from other countries in SSA. Data from Ethiopia and Zambia demonstrated a positive effect of neighborhood diversity on child health outcomes, such as immunization uptake and acute malnutrition [43, 44]. In rural Senegal, an increased social contact among women belonging to two different ethnic groups was indirectly associated with improved child health outcomes through the women empowerment effect [45]. Similarly in Nigeria, high levels of ethnic diversity were found to be predictors of higher utilization of healthcare services [46].

Our study focuses on historically well-established ethnic groups different from ethnic diversity stemming from recent migration, which leads to ethnic minority groupings who are usually very different from the majority population. The main strength of the study was the longitudinal data rich with socio-demographic information, both on an individual and community level permitting control for several variables influencing the relation between ethnic diversity and mortality. Furthermore, indirect standardization enabled adjustment for a wide-ranging list of confounders beside age. Using the Simpson Index to measure diversity allowed a comprehensive look at the composition of the villages and avoided a restriction to minorities only.

Considering the semi-urban town of Nouna which contributes about 30% of the PY as one neighborhood is a limitation of our study. Having all the ethnic groups of the studied population represented in Nouna makes Nouna’s Diversity Index high without considering if these ethnic groups live in isolation. Add to that, Nouna has the lowest SMR in the studied population, which may have led to an over-estimation of the effect of ethnic diversity on mortality. To overcome this limitation, we conducted a sensitivity analysis excluding Nouna town, however the results were almost the same (S2 and S3 Tables).

## Conclusion

Health disparities are determined by individual factors, alongside contextual factors. A number of these factors has been studied and causal pathways have been drawn, however ethnic diversity in relation to health is still under-sought.

Our study showed a connection between historically well-established ethnic diversity in rural areas of Burkina Faso and health. Villages with higher levels of ethnic diversity were associated with lower rates of all-cause mortality.

Generally, the lack of standardization that exists in defining ethnic diversity, as well as in measuring it, alongside the variability of communities, neighborhoods, and villages across and within countries pose a problem to the generalizability of the findings. More research is needed to understand this relation between ethnic diversity and health, to establish it in different settings and to define standardized methods to increase generalizability.

## Supporting information

S1 Checklist Inclusivity in global research. (DOCX)

S1 Table Nouna Health and Demographic Surveillance System sample characteristics from 2000–2012 for villages. (DOCX)

S2 Table Standardized mortality ratios for village characteristics in comparison to the Health and Demographic Surveillance System. Nouna town was excluded. The expected number of cases account for sex, age group, calendar year, and season.
(DOCX)

S3 Table Multilevel Poisson regression of the standardized mortality ratio. Random intercepts were applied on the village level. Nouna town was excluded.
(DOCX)
