# Supplementary material for: Ethnic diversity and mortality in northwest Burkina Faso: An analysis of the Nouna health and demographic surveillance system from 2000 to 2012
Source: PLOS Glob Public Health. 2022 May 6;2(5):e0000267. doi: 10.1371/journal.pgph.0000267 (PMC10021188; doi:10.1371/journal.pgph.0000267)
Supplement: S1 Table — (DOCX) [file pgph.0000267.s002.docx]

| Village | Observed deaths | Person-Years | Crude mortality rate per 1,000 | 95% confidence interval | |
| --- | --- | --- | --- | --- | --- |
|  |  |  |  | lower limit | upper limit |
| Total | 10,137 | 1,016,085.8 | 10.0 | 9.8 | 10.2 |
| Babikolon | 52 | 5,790.7 | 9.0 | 6.7 | 11.8 |
| Bagala | 97 | 12,643.5 | 7.7 | 6.2 | 9.4 |
| Bankoumani | 320 | 23,900.4 | 13.4 | 12.0 | 14.9 |
| Barakui | 99 | 8,516.0 | 11.6 | 9.4 | 14.2 |
| Biron Badala | 29 | 3,469.6 | 8.4 | 5.6 | 12.0 |
| Biron Marka | 61 | 7,194.6 | 8.5 | 6.5 | 10.9 |
| Bissau | 62 | 5,260.6 | 11.8 | 9.0 | 15.1 |
| Boron | 89 | 8,426.3 | 10.6 | 8.5 | 13.0 |
| Bouhuy | 5 | 917.3 | 5.5 | 1.8 | 12.7 |
| Boune | 73 | 8,473.9 | 8.6 | 6.8 | 10.8 |
| Bourasso | 235 | 27,224.2 | 8.6 | 7.6 | 9.8 |
| Cisse | 266 | 14,635.2 | 18.2 | 16.1 | 20.5 |
| Damadigui | 47 | 4,534.6 | 10.4 | 7.6 | 13.8 |
| Dankoumana | 133 | 10,425.1 | 12.8 | 10.7 | 15.1 |
| Dara | 335 | 33,430.6 | 10.0 | 9.0 | 11.2 |
| Dembelela | 68 | 5,920.6 | 11.5 | 8.9 | 14.6 |
| Denissa | 61 | 5,032.0 | 12.1 | 9.3 | 15.6 |
| Mossi | 87 | 9,325.2 | 9.3 | 7.5 | 11.5 |
| Diamasso | 82 | 7,755.7 | 10.6 | 8.4 | 13.1 |
| Dina | 27 | 2,537.0 | 10.6 | 7.0 | 15.5 |
| Dionkongo | 153 | 12,806.4 | 11.9 | 10.1 | 14.0 |
| Dokoura | 71 | 6,149.4 | 11.5 | 9.0 | 14.6 |
| Goni | 493 | 43,474.7 | 11.3 | 10.4 | 12.4 |
| Hinkuy | 16 | 2,076.6 | 7.7 | 4.4 | 12.5 |
| Kamadena | 350 | 29,446.1 | 11.9 | 10.7 | 13.2 |
| Kamiakoro | 88 | 10,892.6 | 8.1 | 6.5 | 10.0 |
| Kansara | 70 | 6,134.8 | 11.4 | 8.9 | 14.4 |
| Kemana | 445 | 32,795.9 | 13.6 | 12.3 | 14.9 |
| Kerena | 59 | 8,399.9 | 7.0 | 5.3 | 9.1 |
| Kodougou | 195 | 19,571.6 | 10.0 | 8.6 | 11.5 |
| Konkuini | 44 | 3,148.4 | 14.0 | 10.2 | 18.8 |
| Koredougou | 18 | 1,670.3 | 10.8 | 6.4 | 17.0 |
| Koro | 342 | 36,248.0 | 9.4 | 8.5 | 10.5 |
| Labarani | 85 | 11,223.2 | 7.6 | 6.0 | 9.4 |
| Lei | 42 | 5,429.8 | 7.7 | 5.6 | 10.5 |
| Lekui | 82 | 13,160.4 | 6.2 | 5.0 | 7.7 |
| Lemini | 65 | 6,433.0 | 10.1 | 7.8 | 12.9 |
| Moinsi | 5 | 861.2 | 5.8 | 1.9 | 13.5 |
| Mourdie | 140 | 13,907.3 | 10.1 | 8.5 | 11.9 |
| Nokui | 133 | 18,323.3 | 7.3 | 6.1 | 8.6 |
| Nouna | 2518 | 315,152.5 | 8.0 | 7.7 | 8.3 |
| Ouette | 195 | 18,580.7 | 10.5 | 9.1 | 12.1 |
| Pa | 179 | 16,200.8 | 11.0 | 9.5 | 12.8 |
| Sampopo | 147 | 11,119.7 | 13.2 | 11.2 | 15.5 |
| Sere | 87 | 8,918.0 | 9.8 | 7.8 | 12.0 |
| Seriba | 245 | 18,935.2 | 12.9 | 11.4 | 14.7 |
| Sien | 40 | 3,191.6 | 12.5 | 9.0 | 17.1 |
| Sikoro | 186 | 15,880.6 | 11.7 | 10.1 | 13.5 |
| Sirakorosso | 21 | 2,457.8 | 8.5 | 5.3 | 13.1 |
| Sobon | 207 | 15,947.2 | 13.0 | 11.3 | 14.9 |
| Soin | 149 | 11,824.5 | 12.6 | 10.7 | 14.8 |
| Solimana | 330 | 26,198.4 | 12.6 | 11.3 | 14.0 |
| Tebere | 96 | 9,386.3 | 10.2 | 8.3 | 12.5 |
| Tissi | 156 | 12,041.8 | 13.0 | 11.0 | 15.2 |
| Toni | 317 | 28,657.6 | 11.1 | 9.9 | 12.3 |
| Tonkoroni | 34 | 2,854.1 | 11.9 | 8.3 | 16.6 |
| Tonsere | 51 | 5,750.3 | 8.9 | 6.6 | 11.7 |
| Zanakuy | 35 | 3,063.9 | 11.4 | 8.0 | 15.9 |
