# Supplementary material for: Ethnic diversity and mortality in northwest Burkina Faso: An analysis of the Nouna health and demographic surveillance system from 2000 to 2012
Source: PLOS Glob Public Health. 2022 May 6;2(5):e0000267. doi: 10.1371/journal.pgph.0000267 (PMC10021188; doi:10.1371/journal.pgph.0000267)
Supplement: S2 Table — Nouna town was excluded. The expected number of cases account for sex, age group, calendar year, and season. (DOCX) [file pgph.0000267.s003.docx]

|  | Observed deaths | Expected deaths | SMR (95% CI) |
| --- | --- | --- | --- |
| Sub-region |  |  |  |
| West | 3,903 | 3,489.5 | 1.12 (1.08 - 1.15) |
| North-East | 2,163 | 1,864.3 | 1.16 (1.11 - 1.21) |
| South-East | 1,553 | 1,734.9 | 0.90 (0.85 - 0.94) |
| Distance to healthcare facility |  |  |  |
| within the village | 2,012* | 2,090.0 | 0.96 (0.92 - 1.01) |
| <5km | 1,493* | 1,362.8 | 1.10 (1.04 - 1.15) |
| ≥5km | 4,114* | 3,636.0 | 1.13 (1.10 - 1.17) |
| Wealth index (fifths) |  |  |  |
| 1^st^ (poorest) | 1,669 | 1,553.3 | 1.07 (1.02 - 1.13) |
| 2^nd^ | 1,941 | 1,714.7 | 1.13 (1.08 - 1.18) |
| 3^rd^ | 974 | 1,021.5 | 0.95 (0.89 - 1.02) |
| 4^th^ | 1,683 | 1,442.5 | 1.17 (1.11 - 1.22) |
| 5^th^ | 1,352 | 1,356.7 | 1.00 (0.94 - 1.05) |
| Religious Diversity (0.00 - 0.64) |  |  |  |
| < 0.36 (less diverse) | 3,367 | 3,055.5 | 1.10 (1.07 - 1.14) |
| 0.36 - 0.49 | 2,240 | 2,086.8 | 1.07 (1.03 - 1.12) |
| > 0.49 | 2,012 | 1,946.4 | 1.03 (0.99 - 1.08) |
| Ethnic Diversity (0.01 - 0.77) |  |  |  |
| < 0.38 (less diverse) | 3,560 | 3,148.0 | 1.13 (1.09 - 1.17) |
| 0.38 - 0.55 | 2,942 | 2,782.4 | 1.06 (1.02 - 1.10) |
| > 0.55 | 1,117 | 1,158.3 | 0.96 (0.91 – 1.02) |
