# Supplementary material for: Ethnic diversity and mortality in northwest Burkina Faso: An analysis of the Nouna health and demographic surveillance system from 2000 to 2012
Source: PLOS Glob Public Health. 2022 May 6;2(5):e0000267. doi: 10.1371/journal.pgph.0000267 (PMC10021188; doi:10.1371/journal.pgph.0000267)
Supplement: S3 Table — Random intercepts were applied on the village level. Nouna town was excluded. (DOCX) [file pgph.0000267.s004.docx]

|  | Exp (coefficient) | 95% CI | P-value |
| --- | --- | --- | --- |
|  |  |  |  |
| level 1 (individual) | |  |  |
| Ethnic group |  |  | <0.001 |
| Bwaba | 0.95 | 0.85-1.06 |  |
| Dafing | 1* |  |  |
| Mossi | 0.78 | 0.71-0.85 |  |
| Peuhl | 0.91 | 0.83-1.00 |  |
| other | 0.88 | 0.77-1.01 |  |
| Religious group |  |  | <0.001 |
| Animist | 1.23 | 1.11-1.35 |  |
| Christian | 0.95 | 0.87-1.03 |  |
| Muslim | 1* |  |  |
| other | 0.91 | 0.57-1.44 |  |
| Level 2 (village) | |  |  |
| Sub-region |  |  | 0.003 |
| West | 1.18 | 1.07-1.30 |  |
| North-East | 1.21 | 1.05-1.40 |  |
| South-East | 1* |  |  |
| Distance to healthcare facility |  |  | 0.001 |
| within the village | 0.87 | 0.80-0.94 |  |
| ≤5 km | 0.95 | 0.87-1.03 |  |
| >5 km | 1* |  |  |
| Wealth index (fifths) |  |  | 0.275 |
| 1^st^ (poorest) | 1* |  |  |
| 2^nd^ | 0.97 | 0.86-1.01 |  |
| 3^rd^ | 1.00 | 0.88-1.13 |  |
| 4^th^ | 1.08 | 0.95-1.24 |  |
| 5^th^ | 0.93 | 0.80-1.07 |  |
| Religious Diversity (0 - 0.64) |  |  | 0.850 |
| < 0.36 (less diverse) | 1* |  |  |
| 0.36 - 0.49 | 1.01 | 0.90-1.14 |  |
| > 0.49 | 1.04 | 0.91-1.18 |  |
| Ethnic Diversity (0.01 - 0.77) |  |  | 0.078 |
| < 0.38 (less diverse) | 1* |  |  |
| 0.38 - 0.55 | 1.06 | 0.96-1.17 |  |
| > 0.55 | 0.92 | 0.82-1.05 |  |
